# A Novel Chitosan/Nano-Hydroxyapatite Composite for the Adsorptive Removal of Cd(II) from Aqueous Solution

**DOI:** 10.3390/polym15061524

**Published:** 2023-03-19

**Authors:** Rachid El Kaim Billah, Ikrame Ayouch, Youness Abdellaoui, Zineb Kassab, Moonis Ali Khan, Mahfoud Agunaou, Abdessadik Soufiane, Marta Otero, Byong-Hun Jeon

**Affiliations:** 1 Laboratory of Coordination and Analytical Chemistry, Department of Chemistry, Faculty of Sciences, University of Chouaib Doukkali, El Jadida 24000, Morocco; 2 Laboratory of Materials and Interfacial Systems, Faculty of Sciences Tétouan, University Abdelmalek Essaadi (UAE), P.O. Box 2121, Tétouan 93000, Morocco; 3 MASCIR Foundation, Rabat Design, Rue Mohamed EL Jazouli, Madinat EL Ifrane, Rabat 10100, Morocco; 4 Faculty of Engineering, Autonomous University of Yucatan, Mérida 97000, Mexico; 5 Department of Sustainability of Natural Resources and Energy, Center for Research and Advanced Studies of the National Polytechnic Institute, Saltillo 25900, Mexico; 6 Materials Science Energy and Nanoengineering Department (MSN), Mohammed VI Polytechnic University (UM6P), Ben Guerir 43150, Morocco; 7 Chemistry Department, College of Science, King Saud University, Riyadh 11451, Saudi Arabia; 8 Departmento de Química y Física Aplicadas, Universidad de Leon, Campus de Vegazana s/n, 24071 Leon, Spain; 9 Department of Earth Resources and Environmental Engineering, Hanyang University, Seoul 04763, Republic of Korea

**Keywords:** nano-hydroxyapatite, chitosan, bio-composite, cadmium, adsorption mechanism

## Abstract

A novel polymer bio-composite based on nano-hydroxyapatite (n-Hap) and chitosan (CS) (CS/n-Hap) was synthesized to effectively address toxic cadmium ions removal from water. The composition and structure of CS/n-Hap bio-composite were analyzed through different characterization techniques. XRD patterns affirmed that the crystalline structure of n-Hap remained unaltered during CS/n-Hap synthesis, while FT-IR spectrum sustained all the characteristic peaks of both CS and n-Hap, affirming the successful synthesis of CS/n-Hap. Adsorption studies, including pH, adsorbent dosage, contact time, initial Cd(II) concentration, and temperature, were carried out to explain and understand the adsorption mechanism. Comparatively, CS/n-Hap bio-composite exhibited better Cd(II) adsorption capacity than pristine CS, with an experimental maximum uptake of 126.65 mg/g under optimized conditions. In addition, the kinetic data were well fitted to the pseudo-second-order model, indicating the formation of chemical bonds between Cd(II) and CS/n-Hap during adsorption. Furthermore, the thermodynamic study suggested that Cd(II) adsorption onto CS/n-Hap was endothermic and spontaneous. The regeneration study showed only about a 3% loss in Cd(II) uptake by CS/n-Hap after five consecutive cycles. Thus, a simple and facile approach was here developed to synthesize an eco-friendly and cost-effective material that can be successfully employed for the removal of toxic heavy metal ions from water.

## 1. Introduction

Environmental pollution induced through rapid technological development is a matter of critical ecological concern. In particular, the contamination by heavy metal ions poses a serious risk to the ecosystem [1]. Among them, cadmium [Cd(II)] is the most concerning heavy metal as it is a cornerstone of recent widespread diseases in developing countries [2].

Cadmium is a rare but naturally occurring element in the earth’s crust, where it is not present in pure state but in combination with other elements (e.g., oxygen (cadmium oxide), chlorine (cadmium chloride), or sulfur (cadmium sulfide)) [3], possibly leaching into ground and surface waters. In addition, significant Cd(II) emissions can be anthropogenically caused by various industrial activities, including metal plating, cadmium–nickel batteries, phosphate fertilizer, mining, pigments, stabilizers, and alloys [4]. Cd(II) is a highly toxic element; excessive human exposure to it can lead to bone degeneration, liver damage, lung failure, hypertension, kidney dysfunction and, eventually, cancer [5].

Adhering to environmental limits and eventually safeguarding the ecosystem reduction in Cd(II) concentration is essential. Various treatment methods, such as chemical precipitation, ultrafiltration, membrane separation, electrochemical deposition, and adsorption, have been engineered in this regard. Although these methods have shown promising results in removing Cd(II) from wastewater, they may present some significant drawbacks, such as high sludge yield, high energy requirements, and secondary pollution generation [6,7]. These shortcomings are avoided by adsorptive methods, which have been proven to be practical in terms of cost, simplicity, versatility, and flexibility. Therefore, adsorption is actually the commercially preferred option for the removal of Cd(II) from water. However, this is conditioned by the choice of a suitable adsorbent based on its specific surface area (SSA), adsorption potential, high availability, and mechanical and chemical stabilities [8,9]. Hence, it is necessary to develop appropriate, efficient, environmentally-friendly and cost-effective adsorbents for Cd(II) removal.

Activated carbon is the most extensively used adsorbent for removing inorganic and organic contaminants from water. However, producing and regenerating activated carbon involves superimposable costs, counting on the precursor raw material and synthesis methodology, which usually involves specific physicochemical processes to refine the adsorbent’s performance [10]. Alternately, synthetic polymers are appealing materials for developing adsorbents by different methods. In addition, their characteristics, such as SSA and chemical functionalities, can be controlled and are highly effective. However, the synthesis approaches are usually complicated and involve specific reagents, which increases net costs [11,12]. In this regard, natural polymers (biopolymers), especially polysaccharides, have received particular attention. Cellulose, chitosan (CS), and alginate are three major natural biopolymers that have been actively employed for the synthesis of materials aimed at the adsorption of heavy metals from contaminated aqueous solutions due to their compelling properties [13,14]. After cellulose, CS is the second most abundant natural polymer. It is widely found in nature and is usually produced by chemical (alkaline) or enzymatic deacetylation of chitin. It has potential applications in biotechnological, agricultural, packaging, pharmaceutical, textile, cosmetic, and many other industries [15,16,17,18,19,20]. As CS is biodegradable, non-toxic, sustainable, and is composed of many amino and hydroxyl groups that can interact electrostatically with heavy metals, it is considered as a valuable material for water treatment [21,22]. However, due to its crystallized structure, CS has poor acid resistance, low porosity, and low mechanical strength [23]. Therefore, CS cannot be used without adequate preparatory modification or combination so to get a chemically stable material in the aqueous phase over a wide pH range, to improve its adsorption performance towards heavy metals by creating more active sites and to enhance porosity. Several research works have been conducted using modified CS as adsorbent for heavy metals. For instance, Sutirman et al. [23] studied divalent heavy metals adsorption on modified CS beads. Wu et al. [24] fabricated phosphorylated magnetic CS/CoFe_2_O_4_ for divalent heavy metal ions uptake. CS-silica hybrid aerogel was developed for divalent heavy metal removal with a maximum uptake of 64.74 mg/g [25]. In addition, Rathinam et al. [26] synthesized a chitosan–lysozyme bio-composite using glutaraldehyde as a crosslinker and investigated its adsorption ability towards Cr(VI). Furthermore, to eliminate Cd(II) and Pb(II) from water, Chen et al. [27] used chitosan/vermiculite bio-composite with an epichlorohydrin (ECH) cross-linking agent, achieving maximum adsorption capacities of 58.5 mg/g and 166.7 mg/g for Cd(II) and Pb(II), respectively, at pH 4.

Nano-crystalline materials generally have a higher SSA and adsorption capacity than conventional materials. In this sense, one of the most promising materials to be combined with CS is nano-hydroxyapatite (n-Hap). N-Hap (Ca_10_(PO_4_)_6_(OH)_2_) is regarded as an environmentally friendly adsorbent due to its biosecurity, low-cost, and excellent storage ability [27,28]. In addition, it displays outstanding biocompatibility and high removal ability towards heavy metals through mechanisms such as ion-exchange, surface complex formation, or dissolution–precipitation. Moreover, the crystalline and porous structure of n-Hap favors the adsorption of metal ions onto n-Hap [27,29]. However, n-Hap nanoparticles tend to aggregate due to the extremely high surface energy induced by van der Waals force, which causes a significant decrease of their SSA and eventually affects their removal capacity [30]. Therefore, the compositing of n-Hap with CS can synergically evolve desired adsorption properties that cannot be attained individually. In the composite, CS serves as a carrier to prevent n-Hap aggregation and increases its adsorption capacities by improving the number of active sites as well as generating functional groups; meanwhile, n-Hap can impart vital chemical and mechanical stability, the potential to immobilize metal cations on its P-OH group sites, and also a large SSA.

In the described context, this work aimed at enhancing the adsorptive performance of the abundant natural polymer CS (extracted from shrimp chitin) by combining it with n-Hap. A new green and innovative CS/n-Hap composite was synthesized, fully characterized, and compared with CS in the removal of Cd(II) from water. A comprehensive assessment of the adsorptive performance under varying experimental conditions such as pH, adsorbent dosage, presence of competing species, contact time, initial Cd(II) concentration, temperature, and competition by counter ions and organic contaminants was accomplished. Kinetic, equilibrium, and thermodynamic parameters were evaluated, and the adsorption mechanism was discussed. In addition, and in view of assessing the sustainable application of the CS/n-Hap composite for wastewater treatment, its reusability was investigated through a cyclic regeneration study.

## 2. Materials and Methods

### 2.1. Chemicals and Reagents

Crude chitin was extracted from shrimp shells waste collected from El-Jadida region in Morocco; calcium nitrate (Ca(NO_3_)_2_,4H_2_O, N° CAS: 13477-34-4), acetic acid (C_2_H_4_O_2_, N° CAS: 64-19-7), diammonium hydrogen phosphate ((NH_4_)_2_HPO_4_, N° CAS: 7783-28-0), nitric acid (HNO_3_, N° CAS:7697-37-2), cadmium nitrate (Cd(NO_3_)_2_, N° CAS: 10022-68-1), sodium hydroxide (NaOH, N° CAS: 1310-73-2), hydrochloric acid (HCl) magnesium nitrate (Mg(NO_3_)_2_, 6H_2_O, CAS N°: 13446-18-9), sodium nitrate (NaNO_3_, CAS N°: 7631-99-4), potassium nitrate (KNO_3_, CAS N°: 7757-79-1), 2-nitrophenol (O_2_NC_6_H_4_OH, N° CAS: 88-75-5), paracetamol (CH_3_CONHC_6_H_4_OH, N° CAS: 103-90-2), and amoxicillin (CH_3_CONHC_6_H_4_OH, N° CAS: 61336-70-7) were purchased from Sigma-Aldrich, St. Louis, MO 63103, USA.

### 2.2. Adsorbent Synthesis

#### 2.2.1. Preparation of Chitosan (CS)

The chitin was treated with a solution of NaOH (48 wt.%) at 100 °C to remove acetyl groups from the chitin, the solid–liquid ratio being 1:20. The so-treated chitin was separated by filtration, washed with deionized (D.I.) water until the solution reached pH ~7, and then oven-dried at 50 °C [31] to obtain the CS to be used in this work.

The CS deacetylation degree was determined by conductometric analysis; 150 mg of CS was completely solubilized in 10 mL of 0.1 N HCl. Thereafter, the volume of the solution was adjusted to 200 mL with D.I. water. The CS solution was titrated with 0.1 N NaOH solution and the solution conductivity was measured after each addition of NaOH.

The degree of deacetylation (DDA) was calculated as:

(1) $$\mathrm{DDA}=\frac{203\;\left(V2-V1\;\right)N\;}{m+42\left(V2-V1\;\right)N}\times 100$$

where N is the normality of the NaOH solution (N), V1 and V2 are the equivalent volumes of NaOH (L), and m is the mass of CS (g). An inoLab™ Cond 7310 Conductometer (Xylem Analytics Germany GmbH, Weilheim, Germany) was used during the analysis.

Additionally, the molecular weight of CS was as well determined by viscometry; one of the most used methods for the determination of the molecular weight of CS is from the intrinsic viscosity η by applying the Marque–Houwink equation as:

(2) [η]= K×M×a

where K and a are constants that depend on the polymer–solvent system at a given temperature, M is the molecular weight in dalton and η is the intrinsic viscosity. The values of K and a are respectively 0.078 mL/g and 0.76 for material solutions prepared in 0.1 M acetic acid/0.2 M sodium acetate at 25 °C.

To measure intrinsic viscosity, a capillary Ubbelohde-type viscometer (0.53 µm) was used. Measurements consisted of the determination (at a given temperature) of the flow time in a vertical capillary tube for the solvent and for the same volume of a CS solution. The photoelectric cells were connected to a digital chronometer giving the flow time in seconds with a precision of 10^−2^ s. Then, the reduction in viscosity was measured for CS solutions at different concentrations. The plot of viscosity versus CS concentration of the solutions results in a line whose intercept is equal to the intrinsic viscosity. From the so-determined value, the M of the prepared CS was calculated. The extracted CS had a M = 345 and a DDA = 93%.

#### 2.2.2. Preparation of Nano-Hydroxyapatite (n-Hap)

The n-Hap was obtained by wet chemical synthesis, as described by Abidi et al. [32]. Briefly, 0.1 M of Ca(NO_3_)_2_·4H_2_O and 0.06 M (NH_4_)_2_HPO_4_ were prepared separately. Subsequently, 100 mL of previously prepared Ca(NO_3_)_2_ solution was transferred into a 500 mL three-neck round-bottom flask and heated at 90 °C for an hour in an oil bath with magnetic stirrer. 100 mL of aqueous (NH_4_)_2_HPO_4_ solution followed by 200 mL of NH_4_OH solution were added dropwise through the left neck of the flask by continuously monitoring the pH of the solution. The solution was continuously stirred for 3 h to obtain the n-Hap powder. The chemical precipitation reaction for the synthesis of n-Hap is presented as:

10Ca(NO_3_)_2_·4H_2_O + 6(NH_4_)_2_HPO_4_ + 8NH_4_OH → Ca_10_(PO_4_)_6_(OH)_2_ + 20NH_4_NO_3_ + 46H_2_O (3)

#### 2.2.3. Preparation of CS/n-Hap Composite

To prepare the CS/n-Hap composite, CS powder (2 g) and n-Hap (1 g) were suspended in 80 mL of a 5% (*v*/*v*) acetic acid solution for 24 h. Then, the solid material was separated by filtration and washed with D.I. water until the solution reached pH ~7. The composite obtained was oven-dried for 12 h at 65 °C. Finally, the composite was manually ground. Figure S1 (Supplementary Materials) schematic presents CS/n-Hap bio-composite synthesis.

### 2.3. Characterization

A Bruker D8 diffractometer was used for X-ray diffraction (XRD) analysis. The sample’s morphology was analyzed by a XL 30 ESEM scanning electron microscopy (SEM) acquired from Philips (Amsterdam, The Netherlands). Active functional groups over the material’s surface were determined by Fourier transform infra-red (FT-IR) spectroscopy using a Nicolet 6700 Spectrometer (Thermo-Fisher Scientific, Massachusetts, USA). Thermal analysis was performed under N_2_ atmosphere using a thermogravimetric analyzer Discovery TGA from TA instruments (Waters Corporation, New Castle, USA). The determination of the point of zero charge (pH_PZC_) was done by the method described by Zaini et al. [33]. Briefly, 0.10 g of material was mixed with 50 mL of 0.01 M NaCl at different initial pHs (pH = 1, 2, 3, 4, 5, 6, 7, and 8). These pH values were adjusted by adding a small amount of 0.1 M HCl/NaOH solutions. The suspensions were allowed to equilibrate for 72 h under mechanical agitation at 25 °C, then centrifuged at 5000 rpm for 10 min, and the final pH of each supernatant was measured using a pH meter inoLab^®^ pH 7310 from Xylem Analytics (Weilheim, Germany).

### 2.4. Adsorption Studies

Cd(II) batch adsorption experiments were done using CS or CS/n-Hap bio-composite as adsorbents. The adsorption experiments were carried out in 150 mL glass beakers containing 50 mL of Cd(II) solutions under stirring at 400 rpm. At equilibrium, residual Cd(II) concentration was analyzed by inductively coupled plasma atomic emission spectrometry (ICP-AES; Thermo Jarrell Ash Corporation Atom Scan 16, Williamston, SC, USA). The limit of detection (LOD) and limit of quantification (LOQ) were respectively determined as 5 and 15 µg/L. The effect of experimental parameters, namely pH, presence of competing species, adsorbent dose, contact time, initial Cd(II) concentration, and temperature on Cd(II) adsorption onto CS/n-Hap was examined. The Cd(II) solution pH (in range 2–8) was adjusted using 0.1 M HCl/NaOH solution, while the CS and CS/n-Hap bio-composite concentration (m) was ranged between 25 and 250 mg, the temperature (T) was varied between 15 and 45 °C, and the initial Cd(II) concentration (C_o_) was varied in range 20–300 mg/L. The contact time (t) during the study was varied in range 5–180 min.

The adsorbed concentration at equilibrium (q_e_, mg/g) and removal percentage were respectively calculated as:

(4) $$q_{e\;}\left(\mathrm{mg}/g\right)=\frac{\left(C_{o}-C_{e}\right)V}{m}$$

(5) $$\mathrm{Removal}\;\left(\%\right)=\frac{\left(C_{o}-C_{e}\right)V}{C_{o}}\times 100\%$$

where C_o_ and C_e_ are the initial and residual Cd(II) concentrations in the solution at equilibrium, respectively. V is the solution volume (L), and m is the mass of adsorbent (g). The adsorbed concentration at a time t (q_t_, mg/g) was calculated using Equation (4) but replacing C_e_ by C_t_ (the residual Cd(II) concentration (mg/L) at time t).

The mechanistic insight during Cd(II) removal was investigated by applying experimental data to adsorption kinetic and isotherm models. In the case of the kinetic study, the used models were pseudo-first order, pseudo-second order and intra-particle diffusion linear models, while for isotherm study, Langmuir and Freundlich models were applied (Tables S1 and S2, Supplementary Materials).

### 2.5. Regeneration Study

During the study, 100 mg CS/n-Hap was saturated with 50 mL Cd(II) solution with a C_o_ of 100 mg/L. The Cd(II) saturated CS/n-Hap was separated and washed with D.I. water to remove unadsorbed traces. Thereafter, the CS/n-Hap sample was separately treated with 50 mL of 0.05 N HCl under stirring for 2 h. Then, the regenerated CS/n-Hap was filtered, washed, and reused for the adsorption of Cd(II). The procedure was repeated for ten consecutive regeneration cycles.

## 3. Results and Discussion

### 3.1. Characterization

#### 3.1.1. X-ray Diffraction Analysis

**Figure 1:**
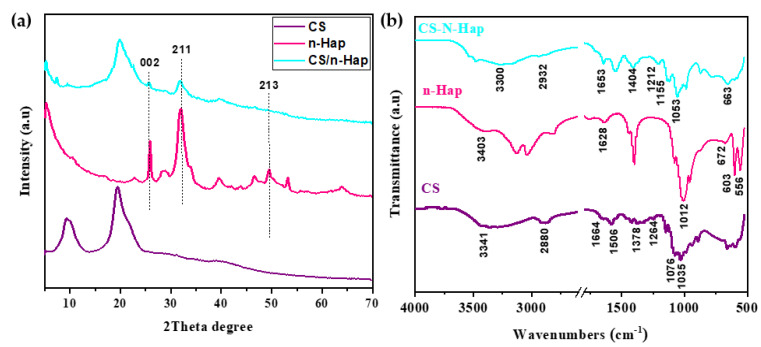
(**a**) XRD patterns, and (**b**) FT-IR spectra of CS, n-Hap, and CS/n-Hap.

The XRD results obtained for CS, n-Hap, and CS/n-Hap composite are illustrated in Figure 1a. The XRD pattern of CS showed two well-defined peaks at 19.4° and 9.6°, corresponding to the characteristic peaks of CS [3,34]. For n-Hap, the peaks at 2*θ* = 25.88°, 31.88°, 32.28°, 34.08°, 39.78°, and 49.58°, corresponding to the diffraction planes (0 0 2), (2 1 1), (1 1 2), (2 0 2), (1 3 0), and (2 1 3) (JCPDS no. 01-073-8417) respectively, confirm the formation of n-Hap [34]. These diffraction peaks were also found in CS/n-Hap composite, at 20.0°, 29.15°, 31.7°, and 33.1°, corresponding to the diffraction planes of CS and n-Hap, respectively [35]. Compositing with CS did not affect the n-Hap crystalline structure, although shorter and broader signals were observed, in line with previous studies [36,37].

#### 3.1.2. Fourier Transform Infra-Red Analysis

A characteristic band at 3341 cm^−1^ was observed in the CS spectrum which corresponds to the stretching vibrations of hydroxyl groups, overlapping with the -NH_2_ stretching vibration peak of CS [35] (Figure 1b). Bands ranging from 1664 to 1506 cm^−1^ can be attributed to the C-O stretching vibrations and the N-H in-plane bending vibrations characteristic of amide I and II structures [35,36], band at 1264 cm^−1^ corresponds to the amide III structure [35].The band at 2880 cm^−1^ was attributed to -CH vibrations, while at 1378 cm^−1^ it was attributed to -CH_3_ and -CH_2_ vibrations. The bands at 1076 and 1035 cm^−1^ correspond to glucosamine stretching vibrations and C–O–C stretching, respectively [35]. n-Hap spectrum displayed bands at 3403, 1628, and 672 cm^−1^ assigned to the stretching and bending of hydroxyl groups [36]. The band observed at 1012 cm^−1^ was assigned to asymmetric stretching, while the bands at 603 and 556 cm^−1^ were assigned to symmetric stretching of phosphate groups [35].

The spectrum of CS/n-Hap composite retained all the characteristic peaks of CS and n-Hap. The band at 3300 cm^−1^ was attributed to -OH groups stretching vibrations; this band decreased in intensity and shifted towards lower wavenumbers in the CS/n-Hap composite due to the intermolecular or intramolecular hydrogen bonds between compounds [38]. The bands at 2932 and 1404 cm^−1^ corresponding to the stretching and bending (–CH) group appeared in the CS/n-Hap composite confirming its formation [35]. The –NH group of CS was included in the 1653 cm^−1^ band [39]. The bands at around 1053 cm^−1^ and 663 cm^−1^ were respectively attributed to the stretching and bending vibrations of the PO_4_^3−^ group present in hydroxyapatite [36,38]. The vibration bands of C–O group were overlapped with phosphate bands at 1150–1040 cm^−1^ [36]. The appearance of the ether bond in the pyranose ring at 1155 cm^−1^ and the amide III band at 1212 cm^−1^ further evidenced the adequate compositing of CS/n-Hap. Moreover, the band broadening around 1053 cm^−1^ showed the presence of the CS polymer and its interaction with PO_4_^3−^ groups of hydroxyapatite [39]. Thus, physical interactions, namely electronic forces and hydrogen bonds between n-Hap and CS may have probably been established during the composite formation [35].

#### 3.1.3. Morphological and Elemental Analysis

Figure S2a,b (Supplementary Materials) displays the SEM micrographs of n-Hap and the CS/n-Hap composite. The morphological images showed that n-Hap particles exhibit a non-uniform shape and size aggregations. Moreover, the CS/n-Hap bio-composite surface displayed a rough and plastic-like appearance. Furthermore, n-Hap particles appeared to be homogeneously dispersed on the polymer surface, indicating good component incorporation [40] and ability of the polymer to bind the solid n-Hap particles [41]. The energy dispersive spectroscopy (EDX) spectra confirm the presence of calcium and phosphate in n-Hap (Figure S2c, Supplementary Materials). Meanwhile, for the CS/n-Hap composite, significant proportions of nitrogen and carbon resulting from the CS structure are evident (Figure S2d, Supplementary Materials).

#### 3.1.4. Thermogravimetric Analysis

**Figure 2:**
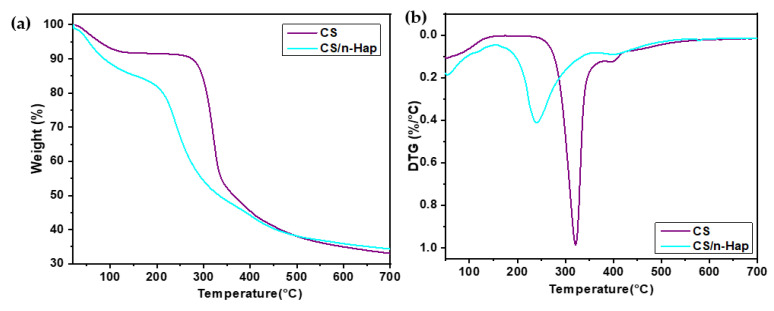
TGA (**a**) and DTG (**b**) plots of CS and CS/n-Hap.

Three main stages of weight loss were observed for both CS and CS/n-Hap samples (Figure 2a,b). The first stage in the range 50–100 °C was attributed to the dehydration of physically retained water, which starts at relative low temperature [41,42]. The thermal decomposition and degradation of the main chain and deacetylation of the CS molecules was responsible for the dominating weight loss at around 228–400 °C in the case of CS [42]. For the CS/n-Hap bio-composite, this weight loss stage occurred at lower temperatures than for CS. This may be due to the improved heat transfer to the CS matrix by the inorganic n-Hap particles present on the surface, leading to rapid degradation of the organic CS [43]. Both CS and CS/n-Hap exhibited other weight loss stage in the range 330–500 °C owing to decomposition of the degraded fragments and slow char oxidation [42]. Finally, residual weight loss at temperatures above 600 °C may be related to the n-Hap dehydroxylation during its thermal decomposition [44].

#### 3.1.5. N_2_ Adsorption–Desorption Isotherm Analysis

N_2_ adsorption/desorption isotherm curves of CS, n-Hap, and CS/n-Hap are depicted in Figure S3 (Supplementary Materials). The isotherms of the three investigated materials were in accordance to type IV isotherms with a H2 hysteresis loop (IUPAC, 1984) [44], which are typical of adsorbents forming aggregates with pores in the shape of plates [45] and suggest that CS, n-Hap, and CS/n-Hap are meso-porous, and that capillary condensation occurs in the pores [46]. Furthermore, compared to CS, which exhibits a SSA of 17 m^2^/g, CS/n-Hap has a higher SSA (about five-fold higher, 87.3 m^2^/g), thus proving that n-Hap incorporation (with SSA 107.5 m^2^/g) enhanced the porous structure of the resulting composite material.

### 3.2. Adsorption Studies

#### 3.2.1. pH Effect, Counter Ions, and Organic Compounds

**Figure 3:**
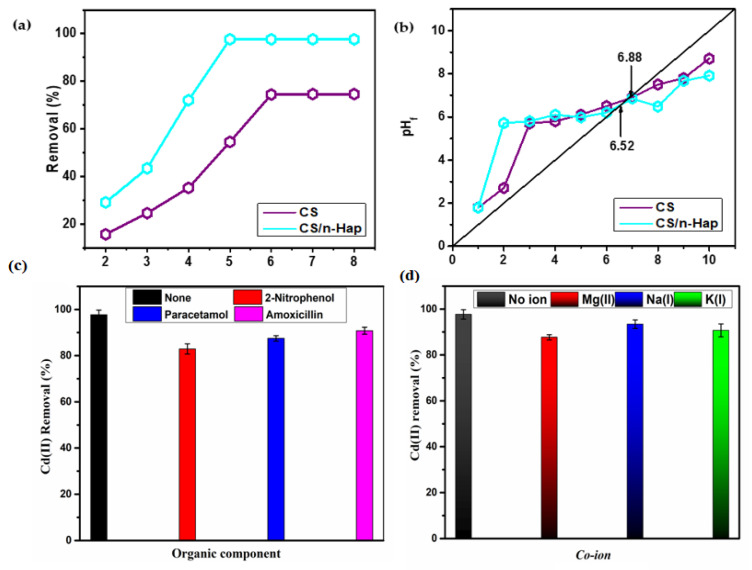
(**a**) Effect of pH on Cd(II) removal, (**b**) point of charge zero (pH_PZC_) plot, (**c**) effect of organic compounds, and (**d**) effect of counter ions on Cd(II) removal onto CS/n-Hap (t: 120 min; m: 2.0 g/L; C_0_: 100 mg/L; T: 25 °C, and counter ions/organic C_o_: 50 mg/L).

The pH of aqueous phase is a parameter that largely affects the adsorption. Figure 3a illustrates that the performance of CS and CS/n-Hap composite towards Cd(II) removal was reliant on the initial solution pH. The maximum Cd(II) uptake on CS/n-Hap and CS occurred at pH 5 and 6, respectively. It was observed that Cd(II) uptake progressively increased from pH 2 to 5 on CS/n-Hap and from pH 2 to 6 on CS, then stabilized with no significant changes at larger pH values. The observed lower Cd(II) uptake under highly acidic conditions must be ascribed to interferences generated by the high concentration of hydronium ions that competed with Cd(II) ions to occupy the active binding sites on the surface of CS/n-Hap and CS, thus inhibiting Cd(II) adsorption [23]. The functional groups involved in binding Cd(II) ions during adsorption were the amine (-NH_2_) groups of CS and the phosphate (PO_4_^3−^) groups of n-Hap. The PO_4_^3−^ groups in CS/n-Hap played an important role during Cd(II) adsorption given the fact that they can display negative charges beyond pH 3. Consequently, as pH increases, the deprotonation degree of these functional groups will also increase and there will be more negative binding sites that allow interaction with metal ions [47]. The pH study results were further supported by the point of zero charge (pH_PZC_) of the investigated materials, which was 6.9 for CS and 6.5 for CS/n-Hap (Figure 3b), denoting that CS and CS/n-Hap surface displayed positive charge at pH < pH_PZC_, while the adsorbent surfaces were predominately negatively charged at pH > pH_PZC_ [48].

The competitive effect of co-existing ions and organic compounds in solution on Cd(II) adsorption was investigated by adding nitrate salts of K^+^, Na^+^, and Mg^2+^ and organic compounds such as 2-nitrophenol, paracetamol, and amoxicillin. Removal percentages of Cd(II) by CS/n-Hap in the presence of mineral salts were higher than 90%. This indicates excellent selectivity of CS/n-Hap for Cd(II) removal in their mineral salts presence (Figure 3c). Meanwhile, the removal percentage of Cd(II) in the presence of organic compounds was slightly lower, although >85% of that in the absence of organic compounds (Figure 3d). Overall, CS/n-Hap showed outstanding selectivity toward Cd(II).

#### 3.2.2. Adsorbent Dose Effect

The effect of CS and CS/n-Hap dosage on Cd(II) adsorption was investigated by carrying out experiments with different masses of these adsorbents, namely in the range 25–250 mg (Figure S4, Supplementary Materials). The results revealed that Cd(II) adsorption percentage on both adsorbents initially increased as their doses increased until reaching their maximum adsorption efficiencies (97.65% on CS/n-Hap and 74.7% on CS). However, increasing adsorbents’ dose did not lead to a significant increase in Cd(II) removal, which indicates that the total available surface area was not enhanced, probably because the adsorption sites were aggregated and/or the adsorbate surface was not fully accessible for adsorption [49].

#### 3.2.3. Contact Time Effect and Kinetic Modeling

**Figure 4:**
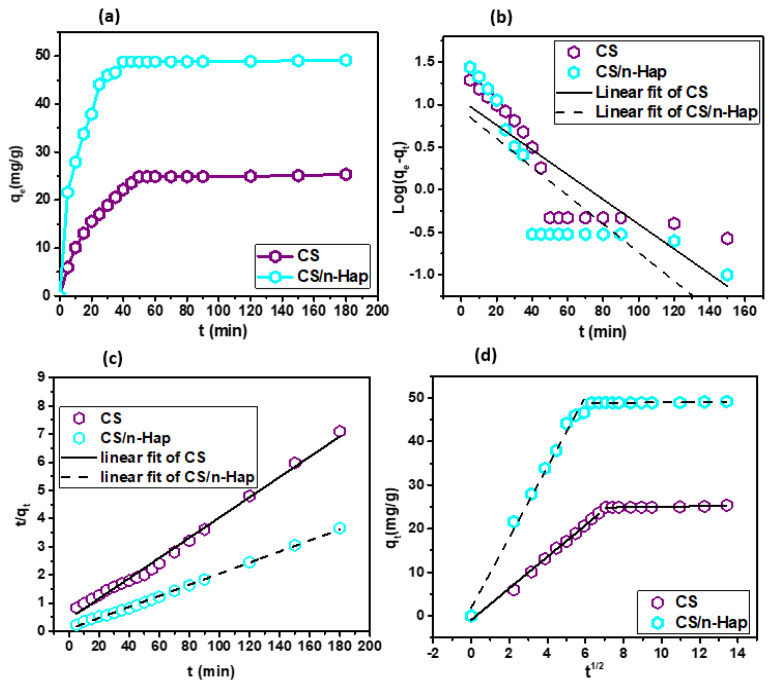
(**a**) Effect of contact time on Cd(II) adsorption, (**b**) linearized pseudo-first order kinetic model fitting, (**c**) linearized pseudo-second order kinetic model fitting, and (**d**) intra-particle diffusion model fitting. (m: 2.0 g/L; pH: 6.0; C_0_: 100 mg/L; and T: 25 °C).

Figure 4a displayed that Cd(II) uptake takes place in two consecutive steps. The first step consisted of a progressive augmentation in the removal percentage, which took about 30 to 50 min for the two investigated adsorbents. The second step corresponded to the surface saturation with Cd(II). However, equilibration time on CS/n-Hap composite was found to be comparatively shorter than on CS, which evidenced the larger efficiency of the composite in the adsorption of Cd(II).

Modeling of kinetic data was done to understand the adsorption dynamics and determine the kinetic rate constant. The pseudo-first order, pseudo-second order, and intra-particle diffusion kinetic models given by equations displayed in Table S1 (Supplementary Materials) were tested to describe the adsorption of Cd(II) onto CS and CS/n-Hap. The linear fittings of the models are illustrated in Figure 4b,c and the kinetic parameters are displayed in Table 1.

The experimental q_e_ values determined for CS (25.36 mg/g) and CS/n-Hap (49.15 mg/g) were nearer to the q_e_ values inferred by the pseudo-second order than by the first-order kinetic model. In addition, the correlation coefficients (R^2^) values for the pseudo-second order model were comparatively higher than those for the pseudo-first order model. Therefore, it can be assumed that the interaction between the two adsorbents and the adsorbate was mainly through chemisorption and that adsorption was mainly controlled by the mass transport rate of the liquid phase or the intra-particle mass transport rate [50]. Intra-particle diffusion rate constants can be obtained from the amount of metal ion adsorbed versus t^1/2^ plots (Figure 4d). The obtained plots presented a double-linearity, indicating that adsorption occurred in two consecutive stages. The first stage corresponds to the external mass transfer (boundary layer diffusion), in which Cd(II) diffuses through the aqueous phase towards the adsorbent. Then, the second stage represents the intra-particle diffusion of metal ions throughout the porous surface of CS and CS/n-Hap.

#### 3.2.4. Initial Adsorbate Concentration Effect and Isotherm Modeling

**Figure 5:**
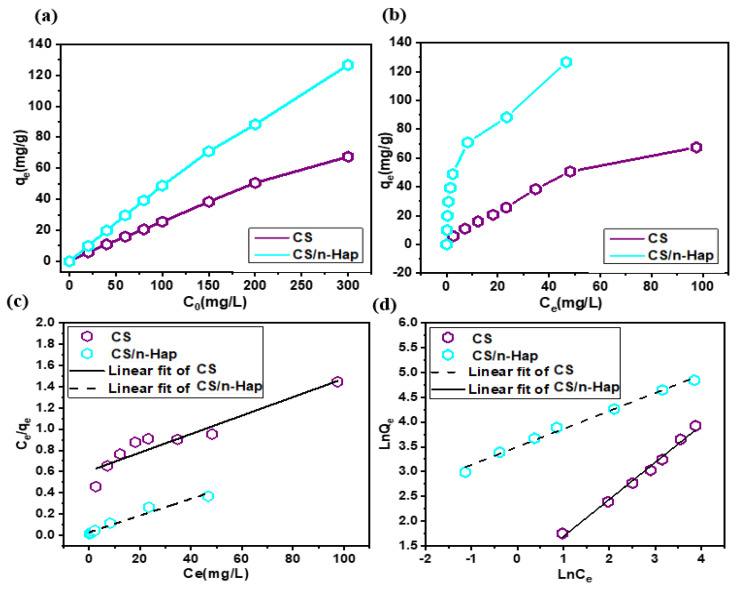
(**a**) Effect of initial Cd(II) concentration on adsorption, (**b**) adsorption equilibrium isotherms, (**c**) Langmuir isotherm model plot, and (**d**) Freundlich isotherm model plot. (t: 120 min; m: 2.0 g/L; pH: 6.0; C_0_: 20–300 mg/L; and T: 25 °C).

As depicted in Figure 5a, the effect of initial concentration on the adsorbed concentration at the equilibrium (q_e_) was significant for the two studied adsorbents. The Cd(II) uptake was notably improved by increasing the initial concentration of the metal ions and practically no saturation was noticed at higher concentrations. Indeed, at the maximum tested initial Cd(II) concentration, namely 300 mg/L, the q_e_ on CS and CS/n-Hap were 67.5 and 126.65 mg/g, respectively. The increase of initial Cd(II) concentration yielded a crucial driving force to exceed all resistances of mass transfer of the adsorbate through the aqueous and solid phases, so favoring its adsorption [51].

The equilibrium concentration of Cd(II) (C_e_) after adsorption onto CS or CS/n-Hap and the corresponding adsorption equilibrium isotherms are plotted in Figure 5b. Both isotherms were positive, regular, and showed an increase in the q_e_ with increasing equilibrium Cd(II) concentration (C_e_), not showing an apparent saturation under the studied experimental conditions, which indicates that there was no formation of a complete monolayer of Cd(II) over the surface of the adsorbents [52].

Linear fittings of experimental data to Langmuir and Freundlich isotherm models presented by equations given in Table S2 (Supplementary Materials) are displayed in Figure 5c,d, which evidence that Freundlich isotherm model gave comparatively better fittings, especially in the case of Cd(II) adsorption onto CS. This was further confirmed by the higher R^2^ obtained for Freundlich than for Langmuir fittings (Table 2). The Freundlich model is associated with adsorbents with heterogeneous surface in which active sites and their corresponding energies are exponentially distributed. Moreover, n > 1 indicates slightly reduced uptake capacity at relatively low equilibrium concentrations. This isotherm model does not predict the adsorbent saturation by the adsorbate; hence, infinite surface coverage is mathematically predicted by the Freundlich model, indicating multilayer adsorption [51]. This agrees well with the observed plot of q_e_ vs. C_e_ for the adsorption of Cd(II) onto CS (Figure 5b).

It should be noted that Cd(II) adsorption performance of CS/n-Hap was better than that of CS, which indicates the favorable effects of compositing n-Hap and CS on Cd(II) adsorption. In the case of Cd(II) adsorption on CS, it was due to electrostatic interaction between the metal ions and the amine groups of CS. Meanwhile, in the case of CS/n-Hap, the amine groups of CS and the phosphate groups of n-Hap synergically provided interaction between these groups and the Cd(II) ions resulting in an improved uptake, as compared with that by CS.

Table S3 [15,25,27,53,54,55,56,57,58] (Supplementary Materials) outlines an overview of the maximum monolayer adsorption capacities along with experimental conditions of different biopolymers, composites, and/or modified adsorbents used for the adsorption of Cd(II) in the literature. The current study demonstrated that CS/n-Hap composite had a good adsorption capacity for Cd(II) in comparison with that of adsorbents in Table S3 (Supplementary Materials).

#### 3.2.5. Temperature Effect and Thermodynamic Modeling

The Cd(II) adsorption on CS and CS/n-Hap was studied at 298, 308, and 318 K and at initial Cd(II) concentration of 100 mg/L. Obtained results (Figure S5a, Supplementary Materials) showed that the adsorption increased with rise in temperature for studied temperature range, with Cd(II) adsorption percentage onto CS displaying a linear trend with temperature. This adsorption behavior may be due to an increase in the mobility of metal ions with rise in temperature. Moreover, the augmentation in temperature can produce a swelling effect in the internal structure of the adsorbents, leading to further infiltration of Cd(II) ions [59].

Thermodynamic parameters, namely the change of Gibbs free energy (ΔG°), enthalpy (ΔH°), and entropy (ΔS°) for Cd(II) adsorption onto CS and CS/n-Hap were determined by applying the van’t Hoff’s law (Equation (5)) to adsorption experimental results obtained at different temperatures [32].

(6) $$\mathrm{Ln}\left(K_{e}^{0}\right)=\frac{\Delta S^{{}^{\circ}}}{R}-\frac{\Delta H^{{}^{\circ}}}{\mathrm{RT}}=-\frac{\Delta G{}^{\circ}}{\mathrm{RT}}$$

where the equilibrium constant ($K_{e}^{0}$) was expressed as [39]:

(7) $$K_{e}^{0}=\frac{1000\times K_{L}\times M\left(\mathrm{Adsorbate}\right)\times \left[\mathrm{Adsorbate}\right]{}^{\circ}}{\gamma }$$

where R is the universal gas constant (8.314 J/K.mol), T is the temperature in K, M(Adsorbate) is the molar mass of the adsorbate, γ is the activity coefficient, and [Adsorbate]° represents the standard concentration of the adsorbate (1 mol/L). ΔH° and ΔS° were calculated from the slope and intercept of the representation of ln$K_{e}^{0}$ versus 1/T (Figure S5b, Supplementary Materials). The magnitudes of these parameters are presented in Table 3. As it may be seen by the parameters in Table 3, the adsorption process of Cd(II) was spontaneous for both CS and CS/n-Hap, and the values of ∆G° were negative at all the studied temperatures. The ∆H° values for both adsorbents were found to be positive, suggesting endothermic process. Furthermore, the positive ∆S° values assume an associated increase in the degree of randomness of the solid/liquid interface, with consequent structural changes in the adsorbate/adsorbent system [53,60].

#### 3.2.6. Adsorption Mechanisms

**Figure 6:**
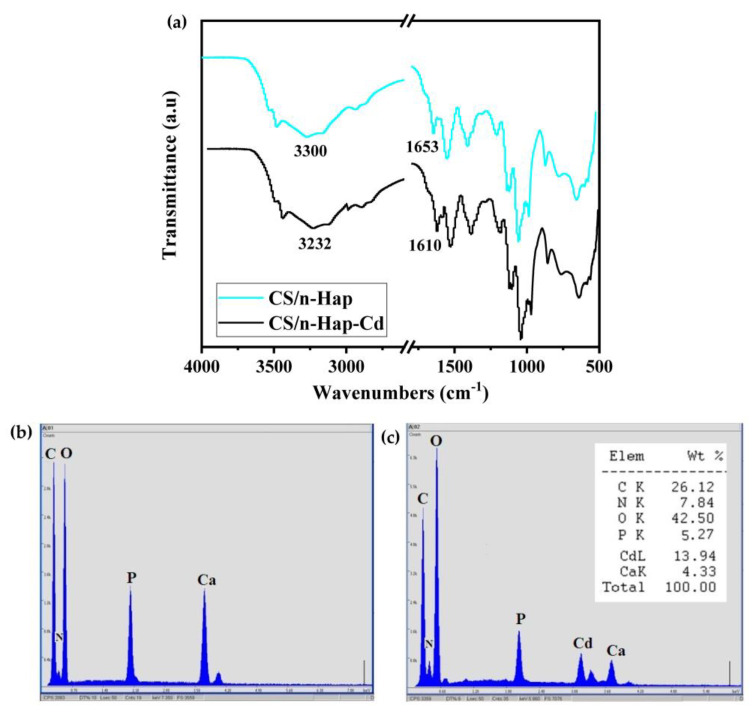
(**a**) FT-IR spectra, EDX of CS/n-Hap, (**b**) before adsorption, and (**c**) after Cd(II) adsorption.

Infra-red spectroscopy and elemental analyses of pristine and Cd(II) saturated CS/n-Hap were performed to depict the mechanism involved during Cd(II) adsorption on CS/n-Hap, illustrated in Figure 6. As presented in FT-IR spectrum (Figure 6a), a band shift corresponding to the hydroxyl groups from 3300 to 3232 cm^−1^ was observed, which could be due to the electrostatic interaction between these functional groups and Cd(II) ions. In addition, adsorption by chelation of Cd(II) ions with the lone electron pair of CS nitrogen occurred due to the shift in the band of –NH group of CS from 1653 to 1610 cm^−1^ [45]. From the EDX analysis (Figure 6b), it was observed that, after adsorption, the Cd(II) percentage on CS/n-Hap was 13.94% and Ca(II) present on CS/n-Hap decreased by 10.39%. Thus, in the case of CS/n-Hap, the adsorption of Cd(II) was essentially governed by ion-exchange mechanism. Since the Cd(II) ionic radius (0.095 nm) is slightly smaller than Ca(II) radius (0.099 nm) [61], the latter can be easily replaced by Cd(II) in the n-Hap crystal lattice:

(8) $${\mathrm{Ca}}_{10}{\left({\mathrm{PO}}_{4}\right)}_{6}{\left(\mathrm{OH}\right)}_{2}+{\mathrm{xCd}}^{2+}\to {\mathrm{Ca}}_{10-x}{\mathrm{Cd}}_{x}{\left({\mathrm{PO}}_{4}\right)}_{6}{\left(\mathrm{OH}\right)}_{2}+{\mathrm{xCa}}^{2+}$$

Furthermore, as stated in the literature, neutral hydroxylated and negatively charged species of Hap (≡POH, ≡CaOH, ≡PO^−^) could as well be involved in the Cd(II) adsorption mechanism according to the surface complexation reactions [62] that are next depicted:

(9) $$\equiv \mathrm{POH}+{\mathrm{Cd}}^{2+}\to \equiv {\mathrm{POCd}}^{+}+H^{+}$$

(10) $$\equiv {\mathrm{PO}}^{-}+{\mathrm{Cd}}^{2+}\to \equiv {\mathrm{POCd}}^{+}$$

(11) $$\equiv \mathrm{CaOH}+{\mathrm{Cd}}^{2+}\to \equiv {\mathrm{CaOCd}}^{+}+H^{+}$$

Hence, we can conclude that the Cd(II) adsorption on CS/n-Hap involves electrostatic interaction, chelation, ion-exchange, and surface complexation mechanisms.

### 3.3. Regeneration Study

**Figure 7:**
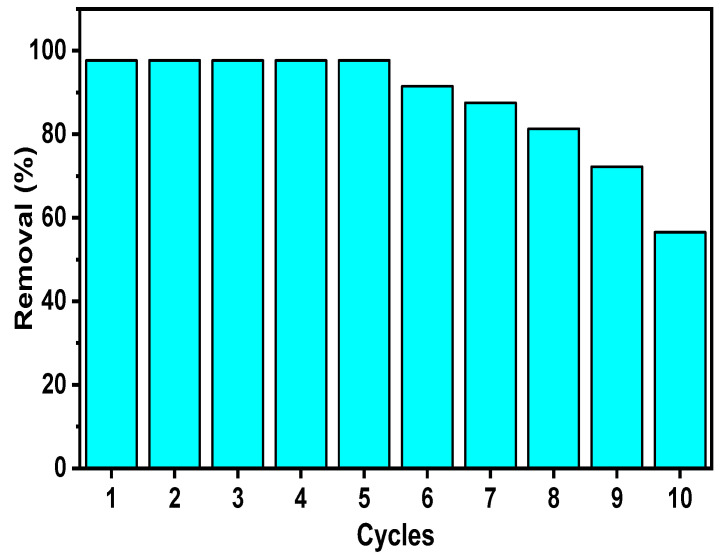
Regeneration-reutilization plot of CS/n-Hap.

The possibility of an adsorbent being easily regenerated and stable through regeneration allows for its reutilization, which is highly valued from an economic point of view. In order to find if this was the case of the CS/n-Hap composite, it was subjected to successive adsorption/desorption cycles. As illustrated in Figure 7, the removal percentage of Cd(II) by CS/n-Hap was initially 97.65%, which still maintained after five regeneration cycles, testifying the great stability and excellent regeneration ability of the here synthesized CS/n-Hap composite. After the fifth regeneration cycle, the adsorption performance of CS/n-Hap progressively decreased, reaching 56.5% after ten successive regeneration cycles due to the decline in active adsorption sites after repeated regeneration cycles [63]. Nevertheless, even after five regeneration cycles, CS/n-Hap was able to adsorb Cd(II). Hence, it was evidenced that this bio-composite can serve as an economical adsorbent for an efficient removal of Cd(II) from contaminated water.

## 4. Conclusions

A CS/n-Hap bio-composite was efficiently synthesized with morphological and physicochemical analysis showing excellent interaction between CS and n-Hap. The incorporation of n-Hap improved not only the SSA and thermal stability of the synthesized composite but also the Cd(II) uptake by the bio-composite, which provided an experimental maximum uptake (128.65 mg/g) that was about twice that of CS (67.5 mg/g). Additionally, the pH study revealed that the functional groups of the composite could be highly affected by the acidity of the medium, in which the higher the pH, the higher the degree of deprotonation of these functional groups will be, and consequently, the more significant the number of negative binding sites directed towards interaction with Cd(II) ions. This finding, along with the infra-red and elemental analyses results after Cd(II) adsorption, actively demonstrated that the adsorption mechanism onto CS/n-Hap was essentially an electrostatic interaction combined with chelation, ion-exchange, and surface complexation. The synthesized material also demonstrated excellent reusability by maintaining high removal percentages (>95%) after five consecutive regeneration cycles. The present study demonstrated that the CS/n-Hap composite could be used as an efficient, green, and cost-effective adsorbent for the removal of Cd(II) from contaminated water.

## Figures and Tables

**Table 1:** Kinetics parameters for Cd(II) adsorption on CS and CS/n-Hap.

Kinetic Model	Parameters	CS	CS/n-Hap
Pseudo-first order	*k_f_* (1/min)	0.1457	0.0167
	*q_e_* (mg/g)	11.24	8.59
	*R*²	0.7221	0.6463
Pseudo-second order	*k_s_* (g/mg.min)	0.0028	0.0045
	*q_e_* (mg/g)	27.85	50.94
	*R*²	0.9923	0.9981
Intra-particle diffusion	*k*_1_ (mg/min^1/2^ g)	3.6242	8.0621
	*C*_1_	0.9386	1.8667
	*R*_1_^2^	0.9947	0.9886
	*k*_2_ (mg/min^1/2^ g)	0.0616	0.0382
	*C*_2_	24.394	48.557
	*R*_2_²	0.7501	0.7589

**Table 2:** Isotherm parameters for Cd(II) adsorption on CS and CS/n-Hap.

Adsorbent	Isotherm Models					
	Langmuir			Freundlich		
	q_max_ (mg/g)	K_L_ (L/mg)	R^2^	K_F_ (mg/g) (L/mg)^1/n^	n	R^2^
CS	115.07	0.0143	0.8941	2.5804	1.3401	0.9915
CS/n-Hap	126.58	0.2743	0.9637	5.818	2.7616	0.9923

**Table 3:** Thermodynamics parameters for Cd(II) adsorption on CS and CS/n-Hap.

Adsorbent	Thermodynamic Parameters				
	ΔG° (kJ/mol)			ΔH° (kJ/mol)	ΔS° (J/mol K)
	298 K	308 K	318 K		
CS	−28.77	−30.75	−32.17	124.73	460.7
CS/n-Hap	−36.33	−40.35	−47.18	31.17	122.66

## Data Availability

Data is available from the corresponding author on reasonable request.

## Supplementary Materials

The following supporting information can be downloaded at: https://www.mdpi.com/article/10.3390/polym15061524/s1, Figure S1: Schematic presentation of Cs/n-Hap bio-composite synthesis; Figure S2: SEM micrographs and EDX analysis of (a,c) n-Hap, and (b,d) Cs/n-Hap bio-composite; Figure S3: N2 adsorption/desorption isotherms of Cs, n-Hap, and Cs/n-Hap; Figure S4: Effect of the adsorbents dosage on Cd(II) removal; Figure S5: (a) Effect of temperature on Cd(II) adsorption onto Cs and Cs/n-Hap, (b) Van’t Hoff plot for Cd(II) adsorption onto Cs and Cs/n-Hap; Table S1: Kinetic models used; Table S2: Used isotherm models; Table S3: Comparison of maximum monolayer adsorption capacities of Cd(II) on various adsorbents. References [59,60,61,62,63] are cited in the Supplementary Materials.
