# Supplementary material for: A Novel Chitosan/Nano-Hydroxyapatite Composite for the Adsorptive Removal of Cd(II) from Aqueous Solution
Source: Polymers (Basel). 2023 Mar 19;15(6):1524. doi: 10.3390/polym15061524 (PMC10058910; doi:10.3390/polym15061524)
Supplement: Supplementary file 1 [file polymers-15-01524-s001.zip › polymers-2062353-supplementary.pdf]

Supplementary Data

Chitosan/nanohydroxyapatite biocomposite for divalent cadmium ions removal from water

Table S1. Kinetic models used.

Kinetic model	Linear equation
Pseudo first order	$log(q_e - q_t) = logq_e - k_1 t$
Pseudo second order	$\frac{t}{q_t} = \frac{1}{k_2 q_e^2} + \left(\frac{1}{q_e}\right) t$
Intra-particle diffusion	$q_t = k_{id}\sqrt{t} + C_i$

k_1 and k_2 are the rate constants for pseudo-first-order and pseudo-second-order kinetic models respectively, k_{id} is intra-particle diffusion constant and C_i measures the thickness of the layer.

Table S2. Used isotherm models.

Isotherm model	Linear equation
Langmuir	$\frac{C_e}{q_e} = \frac{1}{k_L q_m} + \frac{1}{q_m} C_e$
Freundlich	$lnq_e = lnk_F + \frac{1}{n} lnC_e$

q_m is the maximum monolayer adsorption capacity of the adsorbent, k_L and k_F are Langmuir and Freundlich constants, respectively.

Table S3. Comparison of maximum monolayer adsorption capacities of Cd(II) on various adsorbents.

Adsorbent	Experimental conditions	q_{max} (mg/g)	Reference
Ash/GO/Fe_3O_4	m: 1 g/L, t: 1h, pH: 6	47.16	[54]
Sulfhydryl modified attapulgite	m: 1 g/L, t: 2h, pH: 6.5	22.71	[55]
Phosphate sludge/alginate composite	m:1 g/L, T: 25 ºC, t: 3 h, pH: 7	54.27	[53]
Cs-silica hybrid aerogel	t: 6 h, pH: 3	67.57	[25]
Alginate hydrogel	m:1 g/L, T: 25 ºC, t: 3 h, pH: 7	68.15	[15]
Gelatin/clinoptilolite hybrid nanocomposite	T: 25 ºC, t: 1 h	78.13	[56]
Thiol-functionalized mesoporous silica	m: 0.1 g/L, t: 6 h, pH: 7	85.0	[57]
Carboxylic acid-functionalized fibrous Silica KCC-1/polyamide 6 nanocomposite	m:1.67 g/L, t: 4h, T: 25 ºC, pH: 7	109.2	[58]
Hap modified sludge-based biochar	m:1 g/L, t: 24h, T: 25 ºC	114.68	[27]
Cs/n-Hap	m: 2 g/L, t: 1.5 h, pH: 6	126.65	This study

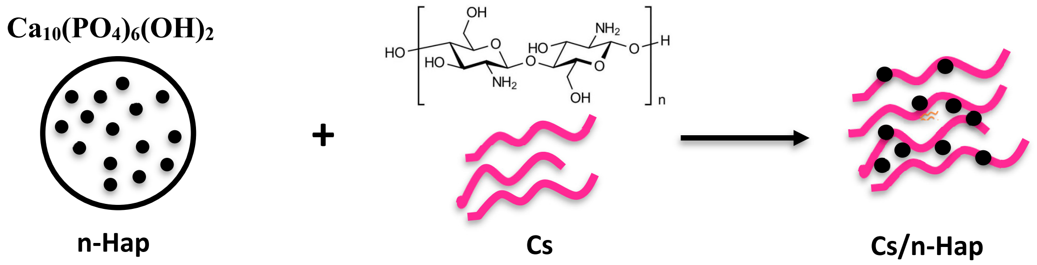

Figure S1. Schematic presentation of Cs/n-Hap bio-composite synthesis.

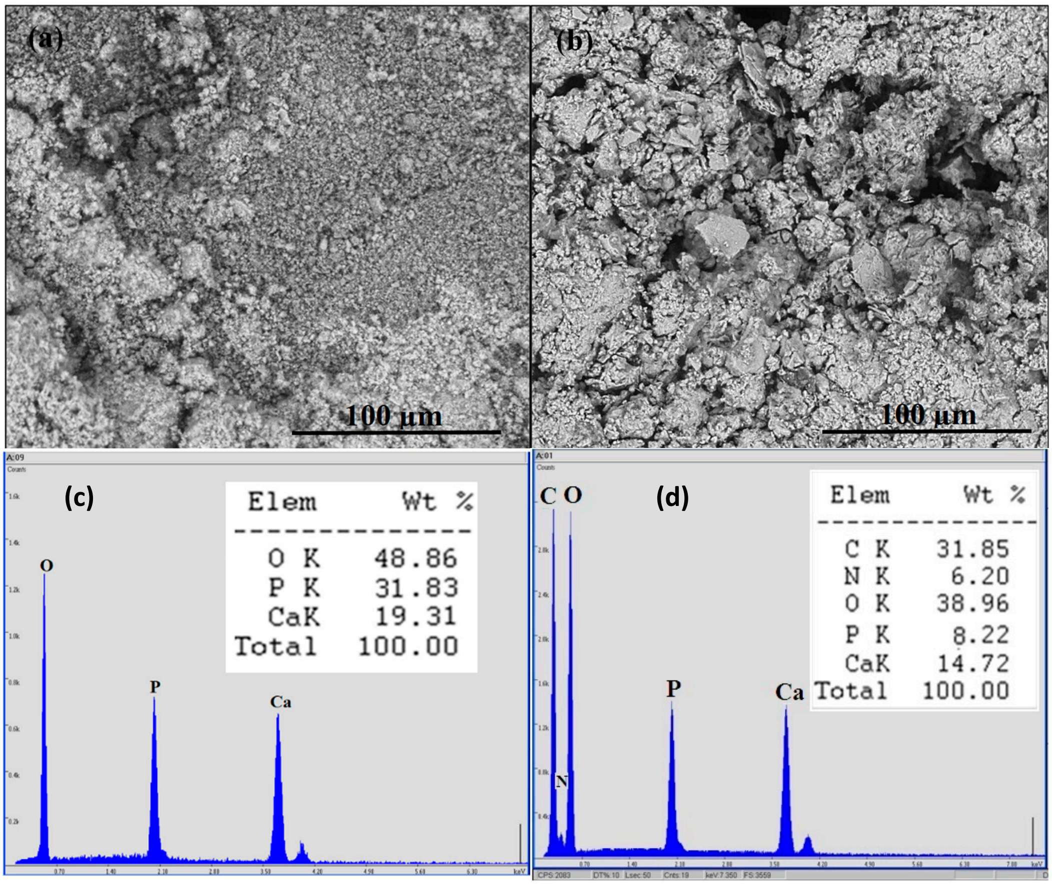

Figure S2. SEM micrographs and EDX analysis of (a, c) n-Hap, and (b, d) Cs/n-Hap bio-composite.

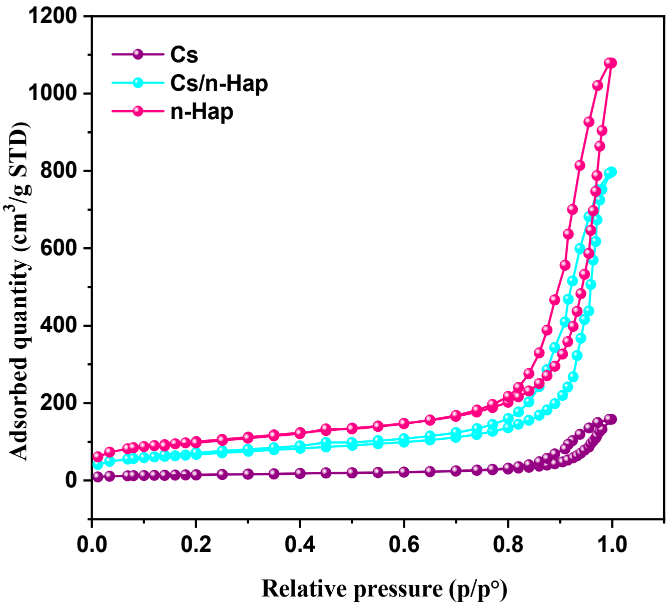

Figure S3. N_2 adsorption/desorption isotherms of Cs, n-Hap and Cs/n-Hap.

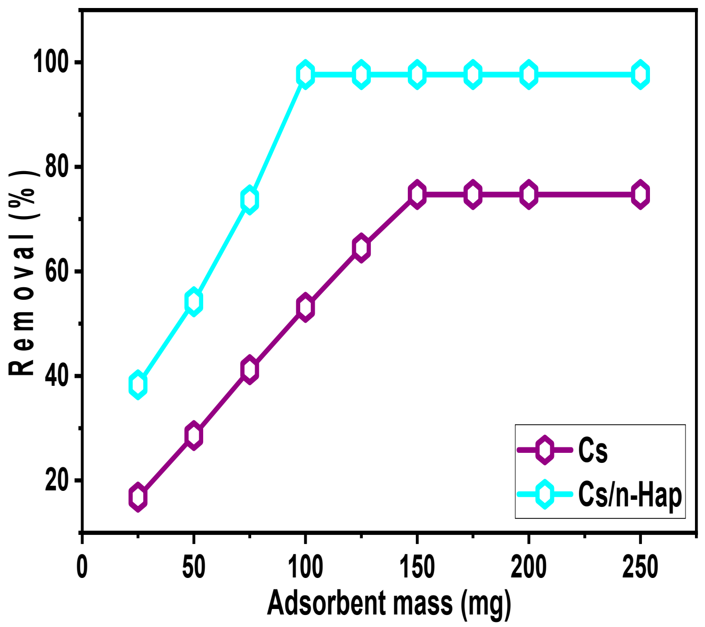

Figure S4. Effect of the adsorbents concentration on Cd(II) removal. **(**Contact time: 120 min; pH = 6.0; C_o = 100 mg/L, and T = 25 °C).

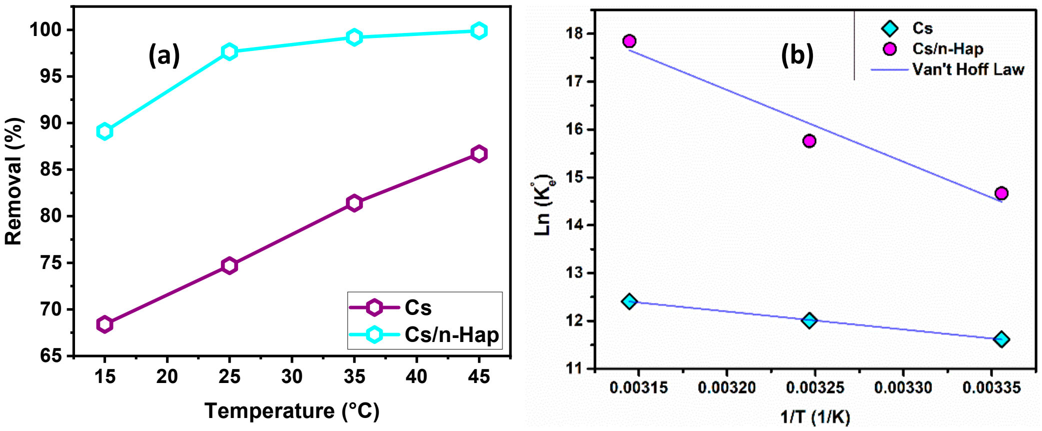

Figure S5. (a) Effect of temperature on Cd(II) adsorption onto Cs and Cs/n-Hap, (b) Van't Hoff plot for Cd(II) adsorption onto Cs and Cs/n-Hap.
